# Surgical Treatment of Shoulder Pathologies in Professional Gymnasts: Findings, Treatment, and Clinical Outcomes

**DOI:** 10.3390/jcm13082183

**Published:** 2024-04-10

**Authors:** Riccardo Ranieri, Matteo Illuminati, Marco Conti, Giacomo Delle Rose, Marco Minelli, Alessandro Castagna

**Affiliations:** 1 Department of Biomedical Sciences, Humanitas University, Via Rita Levi Montalcini 4, 20090 Rozzano, Italy; dr.riccardoranieri@gmail.com (R.R.); marcomariaminelli@gmail.com (M.M.); alessandro.castagna@hunimed.eu (A.C.); 2 Department of Orthopaedics and Traumatology, University Vita-Salute San Raffaele, IRCCS San Raffaele Hospital, Via Olgettina 58, 20132 Milan, Italy; 3 IRCCS Humanitas Clinical and Research Center, Via Manzoni 56, 20089 Rozzano, Italy; conti.marco@gmail.com (M.C.); giacomo.delle_rose@humanitas.it (G.D.R.)

**Keywords:** gymnasts, injuries, shoulder surgery, shoulder arthroscopy, return to training, return to competition, SLAP lesion, shoulder instability, rotator cuff

## Abstract

**Background**: This study aims to investigate the impact of shoulder surgery on professional gymnasts through a case series, analyzing the mechanisms of injury and the shoulder lesion patterns, and reporting the clinical outcomes and return to sport. **Methods**: Twenty-nine surgically treated shoulders in twenty-seven professional gymnasts were retrospectively analyzed. Patients were stratified based on predominant symptoms and anatomical lesions in painful or unstable shoulders. Demographic and injury data, pathological findings, surgical procedure information, and data on time and level of return to sport were collected. **Results**: The average age of participants was 20.2 ± 3.8 years. Acute traumatic onset was reported in 51.8% of cases. Shoulders were categorized as painful in 13 cases and unstable in 16 cases. The most common pathologies were capsulolabral injuries (72%), biceps injuries (48%), and rotator cuff injuries (40%). All of the athletes returned to training within an average of 7.3 months, while the return to competition rate was 56%, achieved in an average of 10.3 months. The sport-specific subjective shoulder value was 84.8% ± 16.6%. Half of the patients who stopped competition reported reasons related to symptom persistence, while the other half reported personal reasons. No significant difference in the return to sport was reported in the cases of painful or unstable shoulder. **Conclusions**: Professional gymnasts requiring shoulder surgery commonly present multiple and complex lesions. Returning to training was possible in all cases; however, the possibility of persisting symptoms and other personal factors which may compromise the return to competition should be discussed with the athlete to give them insights into the possible outcomes.

## 1. Introduction

Artistic gymnastics is a sport that requires grace, strength, flexibility, and balance. It is a discipline that has been part of the Olympic games since 1896 [1]. These athletes’ capability depends on the perfect trade-off between the physical fitness level and the complex technical skills required for each apparatus [2]. The shoulder appears to be an essential joint in artistic gymnastics, as it plays a crucial role in many skills and movements. Gymnasts perform low angular velocity movements (e.g., flexion-extension of the shoulder during the swings in the frontal position on the parallel bars) and high angular velocity movements (e.g., rapid shoulder flexion while rising into a handstand during the upswing in a clear hip circle or rapid shoulder flexion during the first jumping back phase of the back handspring) [3,4]. The long swing is a perfect example of these sophisticated movements, characterized by hyperflexion to extension of the shoulder, as the gymnast passes underneath the bar [5,6].

Consequently, this joint is a common place of injury especially in the male category of the sport, because among the apparatuses present in male gymnastics, there is the bar, the parallel bar, and especially, the rings where the biomechanical movement of the shoulder is subjected to maximal stress [3]. A literature review showed that in female artistic gymnasts, upper limb injuries represented 12.8% to 36% of all injuries, with shoulder injuries ranging from 0% to 4.2%. In male gymnasts, however, upper limb injuries are more common, ranging from 36% to 54% of all injuries, and often involve the shoulders (in up to 19% of all injuries). These injuries are mainly caused by chronic overuse due to repetitive technical movements, followed by acute trauma during violent movements, intense muscle contractions, and frank dislocation [3,7].

In addition, an immediate single diagnosis can be difficult due to the high incidence of combined lesions. As an example, a deep partial supraspinatus tear can be present alone or combined with an anteroinferior labral tear (in the case of shoulder instability) or with a posterosuperior labral tear (in the case of posterosuperior glenoid impingement) [8,9]. SLAP lesions occur at a high frequency in gymnastics, and even though they are well described in overhead throwing athletes, they are less studied in gymnasts and seem to have a different arc of lesion [10,11]. Moreover, even though dislocation and subluxation are commonly observed injuries, both in training and competition, it is important to underline that in gymnasts, inferior subluxation can be considered physiologic since it is necessary for the athlete to perform some of the routines and as such, it is considered part of the training and shall not be overcorrected [9].

Despite numerous studies on gymnastics injuries, focused investigations of the impact of surgery on this complex array of pathologies in this sport are lacking. The present study aims to present a case series of professional gymnasts who required shoulder surgery during their career, analyzing the mechanisms of injury and the shoulder lesion patterns, and evaluating the surgical outcomes in terms of the time and level of sport resumption. Hopefully, this will help clinicians to optimize the treatment strategies of these lesions and to give these athletes advice regarding the possible outcomes.

## 2. Materials and Methods

This is a retrospective observational study of a population of elite professional gymnasts competing at national and international levels. This study adheres to the principles outlined in the Declaration of Helsinki and has received approval from the Institutional Ethics Committee of IRCCS Humanitas Research Hospital. This study follows the recommendation of the STROBE statement [12].

The inclusion criteria for this study encompassed athletes who had undergone shoulder arthroscopic surgery at our institution between 1999 and 2022. The study period was selected to encompass a significant timeframe while aligning with the availability of comprehensive medical records. The athletes needed to meet the following conditions: the presence of shoulder pain and/or functional limitations impeding engagement in sports-related activities, following unsuccessful non-surgical treatments for conditions like rotator cuff disorders, biceps issues, or subtle instability; recurring traumatic glenohumeral instability; first-time instability episode (dislocation or subluxation). No exclusion criteria were applied, except in cases where individuals were unwilling or unable to participate in the study.

Preoperative data were collected using a specific survey including the following information: dominant/injured arm, the nature of symptom onset (gradual onset/injury-related; severity of trauma), injury circumstances (training/competition/other situations), preferred gymnastics discipline (rings, parallel bars, asymmetric bars, etc.), symptoms experienced (pain/apprehension/instability/weakness/other sensations), and the context in which these symptoms arose (rest/exertion/sports/other situations). Additionally, the number of subluxations or complete dislocations in cases of instability, attempts at non-surgical treatments, and instances where surgery was postponed due to athletic season considerations were also recorded.

All preoperative evaluations and surgical procedures were carried out by the senior author (A.C.). Before undergoing surgery, all athletes underwent MRI or MR arthrograms. The arthroscopic treatment was the first choice for both the instability procedures and the painful shoulders. All of the patients were treated under arthroscopy and no open procedures were performed.

Multiple lesions can occur in the same patient. To better stratify the athlete’s pathology, the patients were divided based on the clinical, radiological (with MRI and arthroMRI), and arthroscopic findings into 2 groups (painful or unstable shoulder) according to the pathoanatomic classification of the gymnast’s shoulder proposed by Gendre and Boileau [3,9]. More specifically, regarding the clinical evaluation, gymnasts with mainly self-reported pain during specific shoulder movements were classified into the ‘painful shoulder’ group, while those exhibiting clinical signs of instability were categorized into the ‘unstable shoulder’ group. Furthermore, anatomic lesions were grouped into three bigger categories: rotator cuff disorders (RCDs), capsulolabral injuries, and long head of the biceps (LHB) injuries.

Arthroscopic surgeries were all performed in the lateral decubitus position. As for instability cases, the labrum was mobilized and repaired with bioabsorbable suture anchors, double-loaded with 2 non-absorbable high-strand sutures. MiBa stitches [13] were used in most cases, except for the superior labrum which was repaired with mattress stitches posterior and medial to the biceps anchor and simple stitches anteriorly to the biceps, as described in a previous work [14]. Capsular shifts were performed if required according to the degree of capsular laxity. The glenoid rim was divided into 6 regions, as described by Boileau et al. [15], to better denote the location of the labral injury. Labral tears in zone A or F associated with deep rotator cuff tears (“kissing lesions”) were diagnosed as posterosuperior glenoid impingement (PSGI) [16]. SLAP lesions were classified according to the Snyder classification [17].

Rotator cuff (RC) tendon repairs were performed in a single-row fashion with PEEK or metallic triple-loaded anchors with 2 non-absorbable high-strand sutures using a modified Mason–Allen technique [18]. Rotator cuff tears were classified according to the Snyder classification [19]. The repairs of PASTA (Partial Articular Supraspinatus Tendon Avulsion) lesions were performed trans-tendon or “in situ” if the lesion involved less than 50% of the footprint (arthroscopically evaluated). Otherwise, the lesion was completed, and the tendon was repaired. In the event of minimal lesions (A1 according to the Snyder classification), the tendon was debrided or not even threaded [20]. In cases of an intratendinous uncompleted tear, the tendon was debrided or sutured in latero-lateral fashion. Acromioplasty was not performed routinely but only if an evident and macroscopic acromial spur with severe subacromial space reduction was found.

Biceps problems were addressed with a simple tenotomy in cases of evident and severe delamination of the LHB with bad tissue quality that could compromise an arthroscopic tenodesis. Tenotomy plus soft tissue tenodesis was performed [21] in cases of arthroscopic signs of LHB instability and/or mild tearing of the biceps. SLAP II lesions, in the required cases, were treated with the technique previously described [14].

The diagnosis of the shoulder lesions, considering preoperative and operative information, and the surgical treatments were documented in a standardized database. Arthroscopic procedures were systematically recorded and postoperatively rewatched to reanalyze the data.

Post-surgery guidelines and rehabilitation protocols were supervised by the same specialized rehabilitation doctor (M.C.), adapted to the shoulder lesion and to the surgical management, and tuned on clinical progression. Return to full sports activities was allowed only after complete core stability, full shoulder motion, and force restoration, and not before 3–4 months, i.e., the healing time for the repaired tissue.

Postoperative information was gathered through a combination of methods, including written questionnaires and direct communication via telephone. The duration was recorded, in terms of months, that it took the gymnasts to return to their standard training routines (RTT) and, subsequently, to participate in competitive events (RTC). The subjective shoulder values (SSVs) relative to the sport practice were obtained as a percentage from 0% to 100% [9,22]. In instances where athletes did not make a return to training or competition, a thorough assessment was conducted to pinpoint the reasons behind this decision. This assessment encompassed a range of factors, including any ongoing shoulder-related issues and personal considerations such as age, retirement plans, or other private life circumstances. Furthermore, any persisting postoperative symptoms, complications, occurrences of reinjury (whether related to the shoulder or involving other types of injuries), and any information related to the eventual retirement of the athletes was meticulously documented.

### Statistical Analysis

For our statistical analysis, all patients who met the specified inclusion criteria within the designated timeframe were enrolled. Categorical data were summarized using numerical counts, percentages, and 95% confidence intervals. Continuous data were presented in the form of either mean or median values, along with standard deviations or ranges, all accompanied by 95% confidence intervals. In calculating the statistical significance, the normal distribution method was employed, given that the sample size exceeded 20 participants. The threshold for statistical significance was established at *p* < 0.05.

## 3. Results

### 3.1. Patients’ Characteristics and Preoperative Data

Twenty-seven patients with twenty-nine shoulder surgeries were enrolled. At the time of surgery, all of the patients were professional gymnasts. Most of them were competing at the national level, while ten were competing at an international level (four competing in the world competitions or Olympic games). All of the gymnasts were competing in all events, with six of them specialized in the rings event.

One patient had the operated shoulder injured again and one patient necessitated surgeries on both shoulders due to congenital hyperlaxity, specifically Ehlers–Danlos syndrome.

The mean age at surgery was 20.2 ± 3.8. A total of 6 (7 shoulders) patients were women and 21 (22 cases) were males. The dominant arm was involved in 16 patients (55%). The reported mechanism of injury was acute onset following a trauma or technical gesture in 15 gymnasts (51.8%), happening during competition or training; in the remaining athletes, chronic overuse was identified as the main cause of injury (14 cases, 48.2%). In nine (31%) cases, the athlete reported to have experienced at least one dislocation/evident subluxation. Seventeen (63%) gymnasts tried a conservative approach (physiotherapy and/or corticosteroid) before the surgical options, without resolution of the symptoms.

The main presenting symptoms were pain in *n* = 13 and instability in *n* = 16. In the shoulder instability group, 10 were males and 6 were females. In the painful shoulder group, all athletes were males. Nonetheless, athletes mentioned vague and mixed symptoms in *n* = 21 cases (72%). The demographic and injury data are presented in Table 1 according to painful or unstable shoulder groups. Even though all gymnasts performed all routines, the rings, the parallel bars, and the bars accounted for 13 (62%) of the injuries in males (as female gymnasts do not have parallel bars or rings in their events).

### 3.2. Pathological Lesions and Surgical Treatments

The reported pathologies were capsular/labral injuries (*n* = 21, 72%), LHB injuries (*n* = 14, 48%), rotator cuff injuries (*n* = 11, 40%), cartilage or bone damage (*n* = 9, 31%), and an additional category containing less common findings such as posterosuperior impingement (*n* = 4, 14%) and acromioclavicular arthropathy (*n* = 1, 3%). SLAP II to IV lesions were responsible for *n* = 6 out of 21 (29%) capsular/labral injuries and *n* = 12 out of 14 (86%) of the LHB lesions. Bankart lesions (anterior, anteroinferior, and posterior) were also a common finding, present in *n* = 9 out of 21 capsular/labral injuries (43%). Other findings were GLAD lesions (*n* = 2), diffuse capsular insufficiency and/or MGHL (middle glenohumeral ligaments) insufficiency (*n* = 4), anterosuperior labral lesions (*n* = 3), posterosuperior labral lesions (*n* = 3), and posterior labral lesions (*n* = 2). As for the rotator cuff, all the gymnasts in this group (*n* = 11) had a supraspinatus lesion (A1 to A3), and one gymnast also had a subscapularis lesion. Hill–Sachs lesions were reported in *n* = 3 patients who experienced a dislocation.

Even though a gymnast may have multiple injuries present in their shoulder at the same time, the damage was categorized based on the main issue identified during arthroscopy and the most predominant symptom experienced. The two groups that we established were painful shoulders and unstable shoulders. Pathological lesions and treatments are presented in Table 2 according to the painful or unstable shoulder groups.

### 3.3. Return to Training and Return to Competition

The mean follow-up after surgery was 8.1 ± 6.6 years.

In twenty-nine out of the twenty-nine cases, the athlete managed to return to practice, satisfied with the operation.

The mean time to return to gymnastic-specific training for all gymnasts was 7.3 ± 8.1 months (ranging from 4 months to 4 years).

A total of 15 (56%) athletes (16 cases) were able to return to competition, and 12 (44%) athletes (13 cases) did not return to competitive activity. The mean RTC time was 10.3 ± 2.8 months (ranging from 7–8 months to 18 months). For those who were not able to return to competition, six patients chose to stop the professional activity due to personal reasons (age, job, education, and possible RTC level), while six patients (50%) were not able to RTC because of the persistence of symptoms, including two patients who reported instability symptoms even at rest (one received a diagnosis of Ehlers–Danlos syndrome). RTC was 50% (eight cases) within the unstable shoulder group and 61.5% (eight cases) within the painful shoulder group (*p* = 0.806) (Table 3).

Among the 15 patients who returned to competition, 12 athletes (80%) returned to the same level of competition, or even superior, with 2 competing in the Olympic games and 2 competing in the World Cup. Three (20%) gymnasts’ RTC level decreased in two cases because of the persistence of some symptoms that were limiting the performance. The overall sport-related postoperative SSV was 84.8% ± 16.6 (ranging from 50% to 100%). The sport-related postoperative SSV was 83.8 ± 18.9 in the unstable shoulder group and 86.2 ± 13.9 in the painful shoulder group (*p* = 0.968).

The results divided according to the two groups can be found in Table 3.

## 4. Discussion

This study shows how heterogeneous and complex shoulder pathologies are to treat in elite gymnasts. The initial data indicate that the two main mechanisms of injury are acute trauma and chronic overuse, with the former being slightly more prevalent (51.8%) than the other. This study also shows that a higher number (78%) of gymnasts requiring shoulder surgery are male, consistent with what Caine et al. [7] found in their work. In the male category, there are more events that require the body to be in a suspensory position during the entire length of the routine. This puts maximal stress on the shoulder joint articulation, especially in the movements of maximal flexion/extension and internal/external rotation. These findings are in concordance with the work of Gendre and Boileau [3] that indicated forced flexion with locked hands on the bars or rings as the main mechanism of injury in the shoulders of their participants. This was also reported by De Carli et al. in 2012 [8]. Caraffa et al. [23] reported that the traction force to the intraarticular structure reaches the maximum level during the rings event. Moreover, a paper by Takeuchi et al. [10] proposed how, in gymnasts, the traction force to the LHB during suspension events (i.e., hanging from rings or bars) can exert pressure on the superior labrum through the LHB and could potentially result in the formation of a SLAP lesion that extends both anteriorly and posteriorly. This unique injury mechanism, distinct from that seen in throwing athletes, might explain why gymnasts tend to experience SLAP lesions primarily in the anterior part of their shoulders. Among our participants, SLAP lesions ranging from II to IV (according to the Snyder classification [17]) were found in 12 athletes (44%).

The highly variable range of movements that the body of a gymnast must be able to undergo creates an opening for a wide range of injuries. To regroup these pathologies, the pathoanatomic classification of the gymnast’s shoulder made by Gendre and Boileau [3,9] was used, identifying the two groups of unstable shoulders and painful shoulders.

Similar to Gendre and Boileau [3], in the unstable group, a considerable rate of patients (nine shoulders, 31%) reported an instability episode (dislocation/evident subluxation) during sport activities. In the other seven cases where no evident traumatic event was reported, typical lesions of an unstable shoulder (labral tear (zones C-D-E), capsule insufficiency, and MGHL insufficiency) were found. This finding underlies that the instability issue might also derive from chronic overuse mechanisms. From our data, and looking at the literature, it is also possible to notice various degrees of capsular laxity in gymnasts and a form of acquired joint hypermobility [24]. Gymnasts might not consistently recognize symptoms associated with shoulder instability. They undergo routine stretching exercises for their lower shoulders, which helps them smoothly transition from extension and internal rotation to flexion and external rotation while gripping the bars. This stretching regimen is ingrained in their training from a young age. Consequently, gymnasts often consider a certain level of shoulder laxity as typical and may not always appreciate the possibility of experiencing unintended instability in the joint. Differentiating between the normal morphology of the gymnast’s shoulder and capsular pathology can be challenging. According to other authors [25,26,27,28], instability symptoms without previous dislocation or subluxation may be secondary to progressive stretching of the capsule by overuse and/or muscle imbalance in overhead athletes, a condition described as a minor shoulder instability [25]. This theory also appears valid for gymnasts. Surgeons approaching gymnasts with instability issues should be aware of this pathoanatomical mechanism; it is important to tailor the treatment to allow the gymnast to continue their stretching routine without the persistent instability issue.

In the second group, containing athletes highlighting pain as the main symptom without clear signs of instability, the predominant pathologies included rotator cuff tears, especially at the supraspinatus, SLAP lesions from II to IV (present in nine shoulders; 69.2% of painful shoulders), and two cases of PSGI. Interestingly, signs of PSGI were found in four cases; some of the movements performed in gymnastics can resemble the mechanism of ABER described in throwing athletes and so it might be important for surgeons and trainers to also consider this pathology in this population [9,16].

SLAP lesions were commonly found in this series (12 cases), deserving a specific consideration. The arthroscopic treatment of SLAP type II is still controversial, with the problem of giving persistent shoulder stiffness or pain to athletes [29,30,31]. In this study, a technique developed by the senior surgeon, which allows for a meticulous anatomical reconstruction, was used [14]. This technique allows one to avoid over-tensioning the biceps anchor and the superior labrum, which may lead to residual stiffness and clinical symptoms, leaves the articular aspect of the superior labrum loose, reinforcing the medial side, and stabilizes the middle and superior glenohumeral ligaments by a simple stitch anteriorly. Gymnasts with SLAP II (*n* = 8) treated with this technique had an RTT of 100% (8 out of 8) and 75% (6 out of 8) RTC, with 1 gymnast not able to return to competition for personal reasons. The sport-related SSV was 87.5 ± 14.5. This result was comparable to other studies using different techniques for SLAP II repair [9,10] and might suggest that the anatomical repair of the lesion can be a valid solution to allow these athletes to go back to their preinjury level without persistent pain or the need for a biceps tenodesis. This presents to shoulder surgeons another option to treat this lesion.

In this study, all of the patients were able to return to training, but in only 56% of the cases was the athlete able to return to competition with a sport-related postoperative SSV of 85%. The RCT was lower compared to other studies regarding gymnasts [9,10]. In the study by Takeuchi et al., specifically focused on SLAP lesions, all of the patients returned to sport, but among the questioned patients, 11 out of 19 cases reported a return to sport at a lower level and the sport-related SSV was 76%. In the study by Gendre and Boileau, 91% of the athletes were able to return to competition, and the sport-related SSV was 80. In our study, we could not identify risk factors for the inability to return to competition due to the low number of patients. However, some consideration should be made regarding return to sport. Gymnastics is a highly demanding sport that, at least in Italy, if not at the very elite levels, does not give a sustainable and long career. If surgery is required, return to competition requires a mean of 10 months of rehabilitation and specific training (a similar time was reported by Gendre and Boileau and Takeuchi), which is a considerable setback for an elite athlete. A high standard deviation for RTT and RTC demonstrates that a variation in recovery may be expected and underlines the importance of individual rehabilitation and tailoring treatment plans to the specific needs of each athlete. Probably not by chance, in our study, 50% (six cases) of the gymnasts that did not return to competitions provided reasons related to age, retirement, choice of another professional career outside gymnastics, or the improbability of reaching a satisfactory professional level. Only six cases (one patient who underwent operations to both shoulders received a diagnosis of Ehlers–Danlos syndrome at the time of the second surgery) reported persistent shoulder symptoms, leading to a lower number of possible routine performances and a lower level of competition. Given these considerations, when considering shoulder surgery in this population, the surgeon should discuss in advance with the athlete the challenges in reaching a competitive level again.

In terms of performance outcomes, among the athletes who could resume competitive activities, most athletes who returned to competition (80%) were able to maintain or even surpass their previous level. This highlights the positive impact of shoulder surgery on the ability to perform at a high level in gymnastics. Indeed, two athletes participated in the Olympic Games, and two competed in the World Cup, one winning a gold medal in the floor routine (2022 Mediterranean Games), indicating that successful shoulder surgery can enable athletes to excel at the highest level of competition.

Finally, it is also important to underline that all of these pathologies were present in a young population. The mean age at surgery was 20 years. As reported by Desai et al. [1], elite-level gymnasts tend to specialize in their sport by the age of 12 years, with peak training intensity occurring at 18 years of age, consistent with the increased risk of injury, as a work by Bukva et al. sustains [32]. This is a younger age compared to other overhead sports [33]. Understanding the specific shoulder injuries prevalent in gymnasts is vital for tailoring rehabilitation protocols and designing targeted preventive strategies, also considering their young age.

This paper has some limitations. Firstly, this study has a retrospective design relative to the number of pathologies included and a relatively small sample population. However, it was decided to focus our work only on professional gymnasts who required shoulder surgery, which is a rare population. Secondly, a comparison with gymnasts with similar conditions who only underwent conservative treatment was not conducted, because conservative decisions are often subject to season, economic, or sport society reasons. Thirdly, a validated scale for the shoulder function evaluation was not used, and the specific range of motion was not reported; for these elite athletes, the use of a shoulder scale has limited value, given that the final range of motion must be normal, if not even more, otherwise, the sporting gesture would be impossible. Hence, the return to sport was considered the primary outcome. The strengths of this study include the arthroscopic video analysis, the fact that the patients were operated on by the same surgeon, and that patient interview and evaluation were performed by an independent observer, himself a gymnast and orthopedic resident. This study provides a reliable picture of the surgical outcomes of the most common shoulder pathologies encountered in these athletes. Moreover, it can provide a comparison with the few other works present in the literature and it suggests a new effective method to approach the repair of SLAP II lesions, which is strongly debated within the literature.

## 5. Conclusions

Professional gymnasts requiring shoulder surgery commonly present multiple and complex lesions, and at a particularly young age. Modern arthroscopic techniques allow for the efficient treatment of structural lesions, leading to a return to gymnastics training in all cases, but a return to competition in only 56% of cases, reporting a sport-related SSV of about 84.8%. No differences were reported between cases of painful or unstable shoulders. Factors such as the long time until the return to training and competition, the high functional demand of the sport, and other external aspects (other professional careers, age, and personal reasons) have to be considered when discussing surgical options and relative consequences on the athlete’s professional career.

## Figures and Tables

**Table 1:** Demographic data.

	Unstable (16 Cases)	Painful (13 Cases)
Mean age at surgery (years)	19.8 ± 4.5	20.0 ± 3.1
Sex (M:F)	10:6	13:0
Dominant side	9 (56.25%)	7 (53.84%)
Predominant specialty	4 rings/9 generalist/3 floor	6 rings/2 bar/5 generalist
Injury context	9 acute onset/7 chronic overuse	7 acute onset/6 chronic overuse

**Table 2:** Pathological lesions and treatments according to pathoanatomic classification in unstable and painful shoulders. SP, supraspinatus.

Unstable Shoulder (16)			
***Lesions***			
*Rotator cuff*	*Capsulolabral lesions*	*LHB*	*Others*
Supraspinatus A1 (4) Supraspinatus A2 (1)	B-C-D (1); B-C (2); A-B-C-F (1); C-D (2); C-D-E (1); D-E (2); A-E (1); C-E (1); A-C (1); diffuse capsular insufficiency and/or MGHL insufficiency (4); GLAD (2)	SLAP II (2) SLAP IV (1)	PSGI (2) AC arthropathy (1)
***Treatments***			
SP debridement (4)	Labral repair (12) Capsuloplasty ± capsular shift (4)	SLAP repair (2) SLAP IV regularization (1)	SP and labral debridement (2) Distal clavicle resection (1)
**Painful Shoulder (13)**			
***Lesions***			
*Rotator cuff*	*Capsulolabral lesions*	*LHB*	*Others*
Supraspinatus A1 (3) Supraspinatus A2 (2) Supraspinatus A3 (1) Subscapularis delamination (1)	A-B (2); A-B-F (1); A-F (3); A (2); F (1)	SLAP II (6) SLAP III (2) SLAP IV (1)	PSGI (2)
***Treatments***			
SP debridement SP repair Subscapularis repair	Labral repair (9)	SLAP repair (9) (+ LHB repair with PDS (2); SLAP III regularization (1))	SP and labral debridement (2)

**Table 3:** Return to training and return to competition based on the “shoulder groups”.

	Unstable (16 Cases)	Painful (13 Cases)
Return to training	100%	100%
Mean time of return to sport	8 ± 10.7 (months)	6.2 ± 2.3 (months)
Return to competition	8 (50%)	8 (61.5%)
Mean time of return to competition	9.25 ± 2 (months)	10.6 ± 3.7 (months)
Sport-related SSV	84% ± 19%	86% ± 13%

## Data Availability

Data are contained within the article.
